# Natural Bioactive Products and Alzheimer’s Disease Pathology: Lessons from *Caenorhabditis elegans* Transgenic Models

**DOI:** 10.3390/diseases10020028

**Published:** 2022-05-13

**Authors:** María D. Navarro-Hortal, Jose M. Romero-Márquez, Safa Osta, Victoria Jiménez-Trigo, Pedro Muñoz-Ollero, Alfonso Varela-López

**Affiliations:** Biomedical Research Center, Department of Physiology, Institute of Nutrition and Food Technology “José Mataix Verdú”, University of Granada, Avda del Conocimiento s/n., 18100 Granada, Spain; mdnavarro@ugr.es (M.D.N.-H.); romeromarquez@ugr.es (J.M.R.-M.); safaosta@hotmail.com (S.O.); victoriajt@correo.ugr.es (V.J.-T.); pedrollero@correo.ugr.es (P.M.-O.)

**Keywords:** neurodegenerative disease, beta-amyloid, tau protein, nutraceutical, food

## Abstract

Alzheimer’s disease (AD) is an age-dependent, progressive disorder affecting millions of people. Currently, the therapeutics for AD only treat the symptoms. Although they have been used to discover new products of interest for this disease, mammalian models used to investigate the molecular determinants of this disease are often prohibitively expensive, time-consuming and very complex. On the other hand, cell cultures lack the organism complexity involved in AD. Given the highly conserved neurological pathways between mammals and invertebrates, *Caenorhabditis elegans* has emerged as a powerful tool for the investigation of the pathophysiology of human AD. Numerous models of both Tau- and Aβ-induced toxicity, the two prime components observed to correlate with AD pathology and the ease of performing RNA interference for any gene in the *C. elegans* genome, allow for the identification of multiple therapeutic targets. The effects of many natural products in main AD hallmarks using these models suggest promising health-promoting effects. However, the way in which they exert such effects is not entirely clear. One of the reasons is that various possible therapeutic targets have not been evaluated in many studies. The present review aims to explore shared therapeutical targets and the potential of each of them for AD treatment or prevention.

## 1. Introduction

Alzheimer’s disease (AD) is an age-dependent, progressive disorder affecting millions of people [1]. Although the cause of AD has not been fully elucidated, its histopathological hallmarks include the presence of senile plaques and neurofibrillary tangles (NFTs) composed of Tau microtubule protein in the hippocampus [2,3,4,5]. Some of the therapeutics for AD that have been developed only treat the symptoms. Thus, there is a lack of effective treatments and medications that completely cure or delay progression of the disease. Therefore, more research on both the disease’s causes and its treatment is urgently needed. This, in part, would require adequate animal models [6,7]. Natural products from living organisms, which contain wide and diverse ranges of chemical compounds, can be used as sources of new AD therapeutics, and might even play significant roles in drug discovery [8]. *C. elegans* offers several particular advantages for the investigation of disorders of the nervous system. Thus, the use of *C. elegans* to study the beneficial effects of natural products on AD might provide a promising paradigm. For this reason, the present review aims to collect the main aspects of the pathogenesis and etiology of the disease and the characteristics of *C. elegans* as a model organism and, particularly, as a model of AD. All with the subsequent objective of deepening our understanding of the effects of many natural products on the main AD hallmarks shown by *AD C. elegans* models, and how they exert such effects.

## 2. Alzheimer’s Disease Etiopathology

Neurodegenerative diseases are age-associated disorders leading to the progressive loss of neurons and neuronal dysfunction. AD is a chronic, neurodegenerative disorder that is the most common form of senile dementia, affecting more than 40 million people worldwide [1]. The main clinical manifestations are cognitive dysfunction, memory loss, and abnormal changes in personality [2]. Generally, the first symptoms start to present in the mid-60s, slowly developing and worsening over time [1]. Although the cause of AD has not been fully elucidated, the histopathological hallmarks observed in patients of this disease have been identified: the presence of senile plaques that are formed by the deposition of β-amyloid peptides (Aβs), and NFTs composed of Tau microtubule protein in the hippocampus [2,3,4,5].

Aβs are insoluble peptides consisting of 40–43 amino acids which derive from the sequential proteolytic cleavage of amyloid precursor proteins (APPs) [9] and form deposits in the hippocampus and basal segment. On the other hand, tau protein is a highly soluble protein mainly found in the neuronal axons of the brain [10] that belongs to microtubule-associated protein family. This protein is combined with microtubules and promotes its stabilization and assembly by regulating its phosphorylation level. Due to this role in maintaining microtubule structure and cytoplasmic transport [11,12], the tau protein is involved in synaptic structure and function maintenance [13], as well as in the regulation of neuronal signaling [2]. Under normal conditions, the number of tau-phosphorylated sites is low. The binding of tau to microtubules is negatively regulated at this degree of phosphorylation. However, tau is abnormally hyperphosphorylated in AD patients [2]. The elevated levels of cytosolic hyper-phosphorylation lead to soluble tau self-aggregation into insoluble paired helical filaments and straight filaments that result in the formation of NFTs [14,15,16]. Consequently, microtubules cannot be properly stabilized due to tubulin configuration changes and polymerization capacity loss [16,17], resulting in defective microtubule functioning [18]. NFTs have been evidenced to be responsible for reducing the number of synapses and producing neurotoxicity [19] and cell dysfunction [20]. Moreover, phosphorylated tau proteins seem to be able to spread between cells [21]. This is very important, since small soluble tau can contribute to the spread of pathological tau, but it can also affect neurodegeneration and cognition [22]. On the other hand, Aβ^1–42^ oligomers also are toxic species for cells. Moreover, Aβ in the form of neurotoxic amyloid plaques recruits more Aβ to form insoluble aggregates and induces mitochondrial damage [23], unstable homeostasis, and synaptic dysfunction [24]. In addition, Aβ induces the hypersensitivity of N-methyl-D-aspartate (NMDA) receptors and also disrupts the regulatory control of NMDA activity, causing excitotoxicity and subsequent synaptic injury. This is expected since glutamate is a key neurotransmitter in maintaining synaptic plasticity, acting on the mentioned receptor as well as the α-amino-3-hydroxy-5-methyl-4-isoxazolepropionic acid (AMPA) receptor [25].

Many factors have been associated with AD, such as inflammation, oxidative stress, metabolic disturbances and the imbalance of neurotransmitters [26,27]. Inflammatory reactions and oxidation are consequences of the activation of microglia and/or astrocytes that are induced by changes associated with the accumulation of aberrant proteins. Moreover, it has been reported that Aβ^1–42^ oligomers cause oxidative damage to synaptic membranes [28,29]. The reduction in cholinergic neuron activity is derived from the degeneration of these neurons in the *nucleus basalis* of Meynert, which is a consequence of pro-inflammatory events triggered by the presence of senile plaques and NFTs in the mentioned neurons. The loss of these neurons contributes to deteriorated cognition, but also alters the permeability of the blood–brain barrier, causing the erroneous transportation of metabolites and hampering the removal of senile plaque, worsening the disease condition [30]. On the other hand, a reduced expression of choline acetyl transferase has been found where acetylcholinesterase (AChE) has increased, contributing to the depletion of acetylcholine and the worsening of dementia. AchE also interacts with Aβs, promoting plaque formation [31]. Aβ also binds to the α7- and α4β2-n-acetylcholine-receptor (nAChR) which causes an impairment in synaptic integrity [32]. Moreover, the degeneration of noradrenergic neurons in the *locus coerulus* is also related to cognitive impairment and neurodegeneration. This can negatively affect synaptic plasticity, and therefore learning and memory in an indirect way, since the mentioned processes are enhanced by astrocytes that are present noradrenergic receptors [33]. More recently, a loss of serotonergic neurons from the brainstem and reduced levels of serotonin have been reported in many AD patients [34]. This is very relevant for explaining memory loss, since the serotonergic cortical input from the midbrain raphe nuclei is responsible for cortical plasticity modulation and memory formation [27]. Lastly, synaptic injuries in AD patients have also been linked to the loss of the inhibitory control of the cholinergic and glutamatergic neurons by the GABAergic neurons [35]. In this sense, a 5HT6R antagonist that has been shown to improve cognitive decline by enhancing serotonin levels via GABAergic neurons, also reduces the formation of amyloid plaque since it reduces the gamma-secretase activity without having any effect on β-secretase [35].

Depending on etiology and pathogenesis, several types of this disorder have been distinguished. It can take the form of late-onset AD and sporadic AD, or early-onset and familial AD [36]. Although some cases of familial AD have been associated with mutations in the tau gene [36,37], it is mainly associated with mutations in the APP or presenilin 1 (PSEN1) and 2 (PSEN2) genes [38,39,40]. In contrast, sporadic AD has a complex etiology, involving genetic, environmental, metabolic, viral, and other factors [41]. Among the genetic risk factors for sporadic AD, the strongest is apolipoprotein E (APOE) 4 (APOE4) [42,43], whereas APOE2 has been suggested as a protective factor [44,45,46,47]. In any case, these proteins have been related to oligomer expression and plaque deposition [48,49]. Many hypotheses to explain AD pathogenesis have been proposed, but the most recently proposed pathogenic mechanisms are derived from two fundamental hypotheses: the amyloid cascade hypothesis [50] and the tau hyperphosphorylation hypothesis [51]. According to the amyloid cascade hypothesis, the dimerization of Aβ from APP proteolysis into increasingly large oligomers is responsible for AD. The hydrolysis of APP under the α-, β-, γ-, and η-secretases yields C-terminal fragments under the α-, β-, γ-, and η-secretases by three pathways: the non-amyloidogenic, the amyloidogenic and the alternative processing pathway [52]. In the non-amyloidogenic pathway, APP is cleaved by the proteases α- and γ-secretase into non-toxic peptides. These are neurotrophic and neuroprotective for nerve cells under normal circumstances. In the amyloidogenic pathway, APP is cleaved to CTF-β by β-secretase and then different lengths of Aβ peptides by γ-secretase, including the 42-residue peptide (Aβ^1–42^), which is more prone to aggregation and plaque formation than the 40-residue peptide (Aβ^1–40^) and has stronger neurotoxicity [53,54]. Finally, the alternative processing route occurs under physiological conditions by η-secretase [2]. In recent years, tau has gained much attention. This is, in part, due to the failure of Aβ-targeting treatments in clinical trials, but also because it has been found that cognitive impairments correlate better with tau pathology than Aβ lesions [55,56]. However, the pathogenesis of pathological tau is very complex, and many aspects remain to be elucidated. In addition to hyperphosphorylation, the acetylation and truncation of tau promotes its aggregation and inhibits its ability to bind to microtubules. Moreover, this causes mitochondrial and synaptic dysfunction [57,58,59,60]. Still, it has also been proposed that tau hyperphosphorylation is a protective mechanism against toxic amyloid deposition. The balance of protein kinase and protein phosphatase activity can be responsible for the degree of Tau phosphorylation. It has been suggested that Aβ would activate tau protein kinase 1β, leading to the abnormal phosphorylation of tau protein and the promotion of the formation of paired helical filaments and NFTs, which accelerate the development of tau pathology [28,29,61]. Hyperphosphorylated tau spreads gradually from the post synaptic site (where the formation of senile plaques starts) to the dendrites and the cell body, and finally from the axon to other neurons by intra-axonal connections. This would result in synaptic dysfunction contributing to dementia and neurodegeneration [62].

The previous hypotheses were based on earlier genetic, biochemical, and histopathological evidence and were subsequently strengthened by longitudinal biomarkers, cognitive, and clinical studies. However, whether Aβ accumulation or tau hyperphosphorylation are the causes of AD, or only part of the pathological changes, remains unknown [2]. Alternative pathological mechanisms of AD from different perspectives have begun to be explored more recently. Soluble Aβ oligomers have been suggested to be more toxic than Aβ senile plaques [63], and several findings support this [64,65]. The possible role of soluble Aβ oligomers in AD onset have been emphasized by the “Aβ oligomer pathogenic theory” that suggests that soluble Aβ oligomers are the initiating factors leading to a series of pathological changes in AD [66]. Other hypotheses involving neurotransmitters, oxidative stress, Aβ and tau prion transmission mechanisms, cerebral vasoconstriction, growth hormone secretagogue receptor 1α-mediated mechanisms, and infections have been proposed. All of these are being considered for the development of strategies for AD treatment [2,67].

## 3. Alzheimer Disease Prevention and Natural Products

Currently, available treatments for AD can only temporarily halt or improve the symptoms of the disease, and mainly consist of AchE inhibitors and N-methyl-D-aspartate (NMDA) receptor antagonists. Moreover, they are usually effective only in some mild to moderate AD patients. However, there is a lack of effective treatments and medications that completely cure or delay the progression of the disease. This, in part, would require determining the cause of the disease, since an apparent difficulty is the lack of validated therapeutic targets and adequate animal models [6,7]. As the major goal of AD research is to find therapies that could treat the disease or prevent it from developing, more research on both disease causes and the design of therapies to treat it is needed.

Natural products from living organisms, which contain wide and diverse ranges of chemical compounds, can be used as sources of new AD therapeutics, and might even play significant roles in drug discovery. Hence, the search for bioactive compounds from natural sources to improve health and prevent multiple diseases is already an interesting strategy in the search for new medicinal therapies [8]. Generally, research has focused on detailing the chemical and biological properties of natural products or compounds present in them, but the scientific evidence needed to exploit their potential health benefits is lacking [8]. Therefore, unraveling molecule bioavailability and bioefficacy, and understanding how they promote health, is an important challenge [8].

## 4. *Caenorhabditis elegans* as a Model for the Screening of Natural Products with Health-Promoting Effects

The nematode *Caenorhabditis elegans* is a free-living nematode from temperate soil environments that was adopted as a model organism for developmental biology studies in the 1960s. In comparison with traditional animal models, this organism possesses many advantages that make it an ideal genetic model. These include its small size and short life span of approx. 3 weeks; it being a completely sequenced and well-annotated, but simple genome; and its large progeny production that is a consequence of both sexual and hermaphroditic reproduction. Thus, they are self-fertilizing animals, allowing for the rapid production of offspring. Moreover, it is a transparent organism consisting of 959 somatic cells, 302 of them neurons. All of these are visible with a microscope throughout the life of the animal. In addition to the mentioned biological properties, research using *C. elegans* does not require approval by the Institutional Animal Care and Use Committees, which also facilitates work [68].

Therefore, *C. elegans* is an attractive model as it offers much greater experimental flexibility and control in a better defined genetic and physiological background. However, all these advantages would be meaningless if the model did not present at least some of the relevant biology of the human disease. This is dependent on the aspect of the disease under investigation. Importantly, there is a clear conservation of cellular and molecular functions between these nematodes and many other animals, including humans [69]. Moreover, the short lifespan of *C. elegans* allows both a quick construction of different transgenic models and a rapid assessment of experimental interventions and the role of aging in pathological phenotypes. In fact, more than 3000 mutant strains are available from the Caenorhabditis Genetics Center (CGC) at the University of Minnesota [70].

As mentioned, one of the challenges of research into new bioactive products from nature lies in unraveling their bioavailability and bioefficacy, as well as understanding how these promote health [8]. Therefore, it is important to find an effective and reliable in vivo model for investigating newly described compounds. *C. elegans* offers a promising solution for research into the potential bioactivity and molecular mechanisms of natural products in vivo. Moreover, it is an ideal model for therapeutic target identification. New compounds tested in *C. elegans* are frequently administrated as supplements, although delivery methods vary between studies. The compounds can be applied to bacteria that are ingested, directly spread onto the surface of the nematode growth medium when they grow in plates, or added to the liquid medium [71,72]. Compounds or substances in the medium can enter *C. elegans* via three distinct routes [72,73]. One route is ingestion, which implies that food sources are recognized and selected by the worm thanks to its chemosensory neurons, and taken up by aspiration through the pharynx. The compounds are then absorbed by intestinal cells from the intestinal lumen in less than 2 min, and are distributed throughout the body [72,73]. However, this is a relatively slow process and feeding activity depends on satiety and food availability, and depends on the nervous system, since it is controlled by various neurotransmitters. The second route involves the compounds being absorbed through the cuticle [73,74]. Importantly, the worm cuticle allows for the diffusion of some materials into and out of the body through a permeability barrier established by the hypodermis [74], which facilitates the access of compounds to the target tissues [72]. However, the permeability of the cuticle strongly limits the concentration of the culture medium of certain substances such as dimethyl sulfoxide (DMSO) [75] that, in certain situations, is used to solubilize or prevent the oxidation of some chemicals to be tested. The third route is the worm’s exposed sensory neuronal cilia [72].

Lastly, the simplicity and manipulability of *C. elegans* make it an attractive candidate for high-throughput screening [76]. In this sense, *C. elegans* was the first multicellular organism to have its genome sequenced and it has shown a high sensitivity to RNA interfere (RNAi). This is very useful to identify the molecular mechanisms underlying the action of protective products. Furthermore, the availability of time-proven genetic tools and genomic resources facilitates and simplifies the identification of therapeutical targets. The use of RNAi contributes to make *C. elegans* ideal for high-throughput functional genetic screens [77].

## 5. *Caenorhabditis elegans* as Model of Alzheimer’s Disease

In order to better understand the pathophysiology of this neurodegenerative disorder, in vitro assays and model organisms need to be developed. Despite the use of unicellular organisms and mammalian cells in culture, they lack the complexity found in a living organism or a whole organ, removing the possibility of observing any systemic effects as they would occur in a whole organism. Consequently, these systems are somewhat crude predictors of safety and efficacy. Alternatively, mammalian models have been utilized to determine the cause of AD as well as design therapies to treat it. This is due to the complexity of AD, which makes it necessary to use an organ with such advanced nature as the rodent brain to see a plausible correlation to this neurodegenerative disease in humans. Unfortunately, because they are very expensive and time-consuming, mammals are not amenable to high-throughput genetic or pharmacological screening. In addition, maintaining constant conditions within each experiment, as well as between different research groups, is also very difficult with such complex organisms.

Therefore, invertebrate animals such as *C. elegans* are attractive models as they offer much greater experimental flexibility and control in a better defined genetic and physiological background. In fact, many studies have been carried out on this nematode, confirming that some approaches, prohibitively expensive in mammals, can readily be taken in this type of model. In addition to the many features mentioned in the previous section, *C. elegans* offers several advantages for the investigation of disorders of the nervous system. There is molecular conservation in the neuronal signaling pathways between invertebrates and vertebrates such as dopamine signaling, as well as a diverse range of chemical entities of natural compounds. Moreover, the morphology and synaptic connections of its 302 neurons have been well described [78,79]. Altogether, this facilitates the in vivo screening of morphological or degenerative changes in all neurons, or in discrete subsets of neurons. Thus, the use of *C. elegans* to study the beneficial effects of natural products on AD might provide a promising paradigm.

Nevertheless, some inconveniences considering the etiopathology of human AD can be identified. Despite an APP homologous, named *apl-1*, being present in the *C. elegans* genome [80], it does not naturally form Aβ because it does not contain an Aβ sequence [80], but also because there is no β-secretase in *C. elegans* [81]. On the other hand, a sole tau homologous, the protein PTL-1, has been found in *C. elegans* encoded by the gen *ptl-1* [82], whose loss of function leads to a reduction in viable progeny and touch sensitivity without affecting development [83]. However, wild-type PTL-1 that regulates neuron integrity and longevity [84] has not been reported to aggregate into fibrils. Although it is unlikely that *C. elegans* can completely capture the pathology of AD, there are several models that can be used to assess Aβ- and tau-induced toxicity, which are two crucial hallmarks. All the models are nematode strains that express human pathological proteins constructed for research on human AD since, as mentioned, *C. elegans* does not naturally form senile plaques and abnormal Tau aggregates. These usually present tissue-specific or inducible expressions of Aβ, so transgenic expression results in interpretable and measurable phenotype changes that can be monitored as indicators to study the effects of bioactive compounds on AD prevention or treatment. Moreover, the change does not sufficiently affect viability to prevent recovery or maintain transgenic strains. As expected, this is an important consideration when attempting to model a pathological process in transgenic models. Furthermore, the development of strains that express fusion proteins, combining the green fluorescence protein (GFP) with the mentioned human pathological proteins, allows the visualization of the dynamics of Aβ or Tau aggregation. Importantly, phenotypes found in transgenic models that involve the expression of a heterologous protein could stem from foreign protein expression per se and, thus, are not unique properties of the specific expressed protein. This possibility is usually addressed by testing the effects of the treatments on transgenic strains expressing a non-toxic heterologous protein as it occurs. In fact, each transgenic strain that has been generated has a control strain with a non-toxic heterologous protein. For this reason, most experiments report an absence of effects on the mentioned control strain. More than 3,000 mutant strains are available at low cost by the GCG, which further facilitates the application of *C. elegans* [69]. To conclude, transgenic *C. elegans* models of human AD can be divided into two categories: the Aβ model (Table 1) and the tau model, which were specifically designed and constructed to mimic Aβ and/or tau pathological roles in AD development.

### 5.1. Human Aβ Transgenic C. elegans Models

#### 5.1.1. Muscle Aβ-Expressing Models

For models expressing human Aβ^1–42^ minigenes in muscle cells, animals were generated with constitutive [85] or temperature-inducible expression [86] of the transgene, which usually leads to progressive or rapid paralysis. The first *C. elegans* Aβ model was made by the body-wall muscle-specific expression of Aβ1-42 under the control of the *unc-54* promoter derived from a myosin gene [85]. The best characterized worm with this type of transgene (P*unc-54*::Aβ^1–42^) is the CL2006 strain that presents a constitutive expression of human Aβ that leads to observable intramuscular Aβ deposits with classic characteristics of human Aβ and progressive, adult-onset paralysis. This model is useful for Aβ structure/function and protein interaction studies. However, the paralysis phenotype shows incomplete penetrance and significant variance in the age of onset. This complicates certain approaches of genetic screening, although it is interesting to evaluate the interaction of aging with Aβ toxicity. Furthermore, it may be difficult to disentangle whether protective compounds are modulating Aβ^1–42^ toxicity or aging physiology. Moreover, some attempts to circumvent these limitations have been performed. In these, the Aβ^1–42^ minigene was combined with signal peptide whose expression in body-wall muscle can be up-regulated by environmental factors during larval stages. The best-characterized transgenic strain of this type is CL4176 which was accomplished by engineering a temperature-inducible expression of Aβ^1–42^ in the muscles. For this, the Aβ minigene transcript that was driven by the body-wall muscle-specific *myo-3* promoter (P*myo-3*::Aβ^1–42^) was destabilizing [86]. As a temperature-sensitive mutation in *smg-1* (that participates in the mRNA surveillance system) is also introduced in the genome of this strain, the expression of the Aβ transgene at permissive temperatures is poor. However, the up-shifting of larval worms to a higher temperature (25 °C) inactivates *smg-1*, leading to a significant increase in both Aβ minigene expression and transcript stability. Consequently, the induction of the transgene expression at high temperatures results in a rapid and completely penetrant paralysis, whereas they present wild-type movement throughout their lifespan when raised at 16 °C [87]. Another interesting Aβ model, expressing a Aβ:GFP fusion protein that allows for the visualization of the dynamics of Aβ aggregation, has also been developed [88]. More recently, the CL2337 strain (P*myo-3*::GFP::degron) has been developed, displaying the inducible expression of human Aβ in muscle and a rapid paralysis phenotype after induction. Other strains created for AD research, but less used, have included: CL2010 (P*unc-54*::Aβ^1–40^); CL2010 (P*unc-54*::Aβ^1–40^); CL2109 (P*unc-54*::Aβ dimer); and CL3115 (P*unc-54*::Aβ Met^35^Cys), with the constitutive expression of human Aβ in the muscle. However, the first one did not show SP cleavage, and the other two did not present amyloid formation. An obvious limitation of the models described above is that the Aβ expression is limited to muscle cells, and therefore does not directly capture the neurodegeneration seen in AD, but they do provide an easy means to study Aβ-induced toxicity and protein aggregation [7,89]. Moreover, in most strains, the neurons are recalcitrant to RNAi [90]. As such, some potential “hits” may have been missed due to the insufficient knockdown of the target mRNA.

#### 5.1.2. Neuron Aβ-Expressing Models

Transgenic *C. elegans* expressing Aβ^1–42^ in the neurons have been developed more recently. These were constructed by expressing the signal peptide::Aβ^1–42^ minigene under the pan-neuronal promoter *snb-1* (P*snb-1*::SP::Aβ^1–42^::long 3′UTR). The phenotype more characteristically includes chemotaxis defects and altered movements in liquid [91]. Other measurable deficits affect odorant preference, associated learning behavior, as well as serotonin-related, experience-dependent learning. In fact, one of the most interesting defects in these transgenic worms was a failure to retain associative memory. This was seen when comparing the behavior of worms that were not conditioned with the behavior of those that were conditioned by placing an odorant solution on the lid of the plates for 2 h in a chemotaxis assay with the same odorant [91]. Strains expressing Aβ^1–42^ in the neurons may more accurately represent the consequences of amyloid-induced toxicity seen in AD [7,82,88,92], but the phenotype changes are more subtle than those observed in muscle Aβ^1–42^-expressing strains, making them potentially problematic for high-throughput screening. Then, a transgenic model, in which the transgene signal peptide::Aβ1–42 minigene was expressed solely in 5 glutamatergic neurons by using the promoter from the *eat-4* gene, was developed, but any gross visible phenotypes have been reported [92]. However, in the UA166 strain, an *eat-4*::GFP (P*eat-4*::SP::Aβ^1–42^) construct was used to demonstrate that Aβ expression led to an age-dependent loss of glutamatergic cells, as this allowed the visualization of them. Lastly, a model without the human Aβ gene but with the pan-neuronal expression of APL-1 was generated, which exhibited deficits in olfactory and gustatory learning behavior as well as touch habituation [93], but these were independent of visible amyloid plaque formation. These findings are very interesting since they suggest that higher levels of APP could contribute to the development of AD independent of amyloid peptide aggregation [93].

### 5.2. Tauopathy Transgenic Models

For tau pathology research, different transgenic *C. elegans* strains that express human tau proteins in worm nerve cells have been constructed. Two of them use pan-neuronal promoters [94,95], while the remaining one expressed tau only in touch cell neurons [96]. Uncoordinated movement and age-dependent neurodegeneration have been described in the strains with pan-neuronal tau expression, while only mechanosensory defects were found in animals that expressed tau only in the touch neurons. One of the models with pan-neuronal expression of the transgene, the CL2355 strain (P*snb-1*::SP:Aβ^1–42^::long 3′UTR), has been used in studies investigating AD therapies [97,98]. Unfortunately, it has been suggested that the phenotypes associated with mutant tau proteins are more closely related to frontotemporal dementia or Parkinsonism. Therefore, they may not be appropriate models for tau pathology in AD. Thereafter, another transgenic strain was developed, expressing a human pseudo-hyperphosphorylated tau protein under the pan-neuronal *rgef-1* promoter. This resulted in the exhibition of a progressive age-dependent phenotype of uncoordinated locomotion with axonal abnormalities in inhibitory motor neurons [94], and so has been considered a more representative model for tau modification in AD.

## 6. Effects of Natural Products in *Caenorhabditis elegans* Models of Alzheimer’s Disease

### 6.1. Effects on Aβ Toxicity

The preventive effect of many products from natural sources on Aβ toxicity have been tested in different *C. elegans* strains. The strains most used to evaluate if the products reduced Aβ toxicity were those expressing Aβ in the muscle wall cells, namely the CL2006 and CL4176 strains. The products evaluated in these models (Table 2) included extracts from a large variety of vegetal products. The tested products included plants used in traditional medicine such as: Chinese liquorice (roots of *Glycyrrhiza uralensis* Fisch) [97], velvet bean (*Mucuna pruriens* (L.) DC.), Indian laburnum (*Cassia fistula* L.) [98], *Ginkgo biloba* L. leaf [99], *Glochidion zeylanicum* (Gaertn.) A. Juss. [100], *Terminalia chebula* Retz. [101], moringa (*Moringa oleifera* Lam.) [102], Indian gooseberry (*Phyllanthus emblica* L.) [103], carqueja (*Baccharis trimera* (Less.) DC.) [104] and *Acorus tatarinowii* Schott leaf [105]. Likewise, infused beverages such as different tea (*Camelia sinenesis* (L.) Kuntze) varieties [106], coffee (*Coffea arabica* L.) [107], Greek mountain tea (*Sideritis scardica* Griseb.) [108], rock tea (*Jasonia glutinosa* (L.) DC.) [109], and yerba mate (*Ilex paraguariensis* A.St.-Hil.) [110] were also tested. Some foods or other items used in gastronomy were also evaluated. These included maple syrup [111], fruits such as strawberry *(Fragaria × ananassa*) [112], pomegranate (*Punica granatum* L.) [113,114], guarana (*Paullinia cupana* Kunth) [115] and cranberry (*Vaccinium*) [116], and spices such as holy basil (*Ocimum sanctum*) [117] and turmeric (*Curcuma longa* L.) [114]. Other tested natural products were the extracts of flowers such as red rose (*Rosa x hybrida*) [118], roselle (*Hibiscus sabdariffa* L.) [119], horned violet (*Viola cornuta*), and pansy (*Viola x wittrockiana*) [120]; essential oils from Kushui rose (*Rosa setate × Rosa rugosa*) [121]; Japanese zelkova (*Zelkova serrata* (Thunb.) Makino) [122]; the brown macroalga *Padina gymnospora* (Kütz.) Sond. [123]; and the extract of different parts of ornamental plants such as coconut (*Cocos nucifera* L.) leaf [124] and Chinese arborvitae (*Platycladus orientalis* (L.) Franco) seeds [103].

Furthermore, a considerable number of assays testing the effects of individual chemical compounds isolated from natural sources in similar models are present in the scientific literature (Table 3). Many of them were isolated from the products described above, including β-citronellol and geraniol from Kushui rose essential oil [121], punicalagin, methyl-urolithin A and urolithin A from pomegranate extract [114], α-bisabolol from *P. gymnospora* [123], dehydroheliobuphthalmin and savinin from *P. orientalis* seeds [103], hydroxycitric acid from *H. sabdariffa* [119], bilobalide B and ginkgolide C from G. biloba leaf [99], 7-hydroxycalamenene from Japanese zelkova essential oil [122], major silymarin components (silybin A, silybin B, 2,3-dehydrosilybin A, 2,3-dehydrosilybin B) [126], bioactive peptides from “barquillo” [125], and several compounds isolated from Chinese liquorice root including glycyrrhetinic acid, liquiritigenin, isoliquiritigenin and glycyrrhizic acid and its aglycon [97]. Other compounds were isolated from non-tested products, including several sequisterpenoids (nardochinin A, B, C and D) from the underground parts of *Nardostachys chinensis* Batal ethanolic extract [133], sesamin, sesamol and sesamolin from sesame (*Sesamun indicum* L.) seeds [134], oleuropein aglycone from extra virgin olive oil [135], magnolol from *Magnolia* genus especies [136], several sesquiterpene lactones (salcastanins, nubiol, nubdienolide, and nubenolide) from *Salvia castanea* [137], otophylloside B from *Cynanchum otophyllum* L. [138], betalains from red-beet (*Beta vulgaris* L.) [139], gossypol from cotton plant (*Gossypium arboreum* L.) [140], cyclotides from butterfly pea [141], resveratrol from red wine [142] and withanolide A from the root of ashwagandha (*Withania somnifera* (L.) Dunal) [143]. Likewise, some well-known bioactive molecules that can be found in multiple sources such as quercetin [144,145], ferulic acid [146], caffeine [110], morin [140], gallic acid, ellagic acid [114] and caffeic acid [147] were also tested in the mentioned assays.

Most of the tested products showed protective effects on the phenotype of the mentioned strains (i.e., they delayed paralysis induced by Aβ toxicity or reduced the number of paralyzed worms), except for pomegranate [114] and red rose extract [118]. In the same sense, other researchers reported the increased survival or lifespan of CL2006 nematodes when they were treated with ferulic acid, quercetin, gossypol, morin [140], caffeine, yerba mate extract [110], oleuropein aglycone [135] and otophylloside B [138]. When isolated compounds were tested alone, the lack of effect on this phenotypic change was more frequent. These included gallic acid, ellagic acid [114], β-citronellol [121], dehydroheliobuphthalmin, savinin [103], punicalagin, methyl-urolithin A, urolithin A [114], silybin A [144], nubdienolid, nubdienolide, nubenolide [137] and several compounds isolated from *G. biloba* leaf extract including flavonoids, bilobalide B, ginkgolide C [99], and nardochinin C [133]. Likewise, no effect was found for a combination of geraniol and β-citronellol isolated from rose [121], although geraniol alone showed healthy effects. Moreover, some compounds, such as epigallocatechin gallate, whose anti-AD activity was clearly established in early studies, have been used as positive controls in more recent studies.

Some of the mentioned and other additional products have also been tested in the CL2355 strain, reducing chemotaxis behavior, olfactory learning and/or the cognitive defects induced by Aβ aggregates. These were ginkgolide A [99], *G. zeylanicum* extract [100], tiger milk mushroom extract [131], cyclotides from butterfly pea [141], and otophylloside B [138]. Other authors have reported that sensitivity to exogenous serotonin was improved by ferulic acid [146], caffeic acid [147], *A. tatarinowii* Schott oil [105], ginkgolide A [99], sesamin, sesamolin [134], Kushui rose essential oil and geraniol (both isolated from Kushui rose essential oil) [121] and β-citronellol [121]. However, red rose [118] extract, sesamol [134] and the rest of the compounds isolated from *G. biloba* leaf extract, ginkgolide B, C and J, bilobalide B and flavonoids [99] had no effect on chemotaxis.

### 6.2. Senile Plaque Formation and Accumulation

Strain CL2006 and, to a lesser extent, CL2355 and CL4176, have also been used to evaluate the effects of selected compounds on fibrillar amyloid formation. All the products that prevented or reduced phenotypic changes in the transgenic models also decreased deposits or plaques of Aβ, at least in the studies where they were measured. Extracts of Kushui rose [121], Greek mountain tea [108], Chinese liquorice [97], and the leaves of coconut [124], *G. biloba* [99] and *G. uralensis* [97] reduced Aβ deposits in the CL2006 strain. A similar effect was reported for several compounds isolated from *G. uralensis* extract including glycyrrhetinic acid, isoliquiritigenin, glycyrrhizic acid and its aglycon [97], as well as ferulic acid [146], otophylloside B [138] and oleuropein aglycone [135]. This protective effect was also reported for extracts of strawberry [112] and the brown macroalga *P. gymnospora* [123] in the CL4176 strain. Likewise, *A. tatarinowii* Schott oil [105] and the extracts of coconut leaves [124] and Greek mountain tea were tested for both CL2006 and CL4176 strains, where they reduced the deposits in both strains. Aβs were also studied in GMC101 worms where magnolol also reduced them [150]. Furthermore, anti-AD activity of the tested products was established in a few studies, solely by investigating the effect on Aβ deposits, meaning that the possible effect on phenotypic changes was not evaluated. This was also the case for the investigations of cyclotides applied to CL2006 worms [141].

Additionally, Western- or immuno-blotting allowed for an inspection to see if the number of deposits or plaques was a consequence of changes in the oligomerization of Aβs. In most of cases, results suggested a lower oligomerization in the treated animals. Aβ oligomerization in CL2006 and CL4176 was decreased by treatments with ginkgolides A and J [99], ferulic acid [146], 2,3-dehydrosilybin A [126], oleuropein aglycone [135], Kushui rose essential oil [121] and the extracts of *G. biloba* leaf [99], cranberry [116] and *T. chebula* [101]. However, the treatment with sesamolin, sesamol [134] and silybin A and B [144] had no effect on oligomerization, despite the reduced amount of Aβ deposits being detected in animals treated with silybin B.

Other studies evaluated the effect of the treatments on the expression of Aβ transgenes. In the expression of *amy-1*, the Aβ transgene was down-regulated by *P. gymnospora* extract and its compound α-bisabolol in CL4176 worms, which correlated with lower levels of Aβ deposits [123]. Aβ transgene expression was also down-regulated by caffeine [110] and otophylloside B [138] in the CL2006 strain, whereas it was up-regulated with sesamol treatment [134] in the CL4176 strain. Notwithstanding, sesamin and sesamolin had no effect on Aβ transgene expression. Curiously, a mixture of (+)-catechins, caffeine and procyanidins had no effect on Aβ transgene expression [130], although they decreased Aβ proteins.

### 6.3. Effect on Toxicity Derived from Tau Aggregates

Compared to the previous amyloidogenic models, the use of transgenic *C. elegans* models of tauopathies is less diffused. In fact, only curcumin, resveratrol and trehalose have been tested in these types of models. The mentioned compounds were effective in reverting the behavioral abnormalities in worms expressing wild-type or R406W-mutated tau at a pan-neuronal level. Curcumin, resveratrol and trehalose have shown protective effects on the mentioned strain. Among them, curcumin was found to be the most effective compound, although the effect was not linked to a reduction in tau expression, phosphorylation or insoluble aggregate formation [79].

### 6.4. Oxidative Stress

High doses of reactive oxygen species (ROS), without the increased activity of antioxidant systems, lead to oxidative stress. The primary source of ROS in most cells is the mitochondrion, where they are generated as a by-product during oxidative phosphorylation, which tends to occur at a higher extent when there are inefficiencies in oxidative phosphorylation. Oxidative stress causes oxidative damage to lipid membranes, and DNA. Furthermore, it is known to disrupt proteostasis when it causes oxidative damage to proteins. The loss of function associated with the misfolding and aggregation of damaged proteins has been related to disease susceptibility. Moreover, the mitochondrion is the major target of ROS. Oxidative damage to mitochondrial components, particularly mitochondrial DNA, could affect oxidative phosphorylation efficiency and energy production [153]. Increased protein carbonyl content [154], neuronal lipid peroxidation [155] and increased ROS levels [99] have been reported in transgenic worms expressing Aβ. Furthermore, mutations in the sel-12 gene that encodes for presenilins in *C. elegans* [156], which are related to the excessive production of Aβ1–42 in early-onset AD, can lead to cell death via oxidative stress [157]. In addition, pathogenic protein aggregation in cells has been associated with an excessive production of ROS because of their effects in proteins, such as amino-acid side chain oxidation, hydroperoxide formation, the carbonylation of proteins and the fragmentation of the protein backbone [158]. This would suggest that human Aβ1–42 minigene expression could cause oxidative stress. In turn, oxidative stress could facilitate Aβ oligomerization, deposit formation, accumulation and, consequently, other phenotypic changes attributed to Aβ toxicity. In fact, an elevation of oxidative stress markers prior to Aβ deposition and senile plaque formation has been reported in CL4176 nematodes, suggesting that this condition is involved in the pathogenesis of AD [159,160].

The treatment with extracts of strawberry [112], guarana [115], roselle [119], Indian laburnum [98], horseradish tree [104], yerba mate [110] and “barquillo” (in both forms, hydrolyzed and not) [125], as well as various isolated compounds including caffeine [110], phosphatidylserine [161], 2,3-dehydrosilybin A/B [126] and withanolide A [143] reduced basal ROS levels in wild-type nematodes. In parallel to this, many studies also incorporated different assays to test the resistance to oxidative stress in the individuals treated with the above extracts (except for the extracts of strawberry and roselle). Most of the tested products increased survival rates in worms stressed by either paraquat [143,147], juglone [98,110,122,126] or H_2_O_2_ [115,125,161]. Moreover, an increased resistance to juglone-induced oxidative stress was reported for nematodes treated with heartwood extract [122] and rock tea [109], despite basal levels of ROS not being measured. The same occurred with carqueja extracts [104] and different tea types [106], in this case, against oxidative stress induced by tert-butyl hydroperoxide [104] and Cr^6+^ [106], respectively. The unique extract that failed in increasing the resistance to chemically induced oxidative stress was guarana extract, but only when the chemical that was used was tert-butyl hydroperoxide [115]. Curiously, guarana extract [115] was able to reduce the increase in ROS intracellular levels after incubation with hydrogen peroxide, which suggests that the pro-oxidant agents used in oxidative stress resistance assays must be considered when results of different studies are compared. A similar effect on ROS increase has been also confirmed in treatments with caffeic acid [147]. Likewise, G. biloba leaf extract decreased juglone-induced intracellular ROS levels [99] and caffeic acid prevented ROS level increase induced by paraquat [147]. Importantly, the effect on ROS levels with guarana [115], yerba mate extract and caffeine [110] were also confirmed to occur in the CL2006 strain, where the effects of kaempherol, quercetin, ascorbate [106], the extracts of G. biloba leaf [99], Tiew kon [127], and P. gymnospora [123], and the constituent of the latter, α-bisabolol [123], on redox biology were not evaluated. Similarly, the treatment with caffeic acid [147], 7-hydroxycalamenene [124], P. gymnospora extract, α-bisabolol [123], G. biloba leaf extract and its component ginkgolide A [99], cyclotides [141], and a mixture of (+)-catechins, caffeine and procyanidins [130] reduced basal ROS levels in the CL4176 strain. Ginkgolide B and C, flavonoids and bilobalide B had no effect on intracellular ROS levels, which correlated with the lack of effect on Aβ-induced paralysis. An exception was ginkgolide J that reduced Aβ-induced paralysis but had no effect on ROS levels. Interestingly, treatment with oleuropein aglycone, which delayed Aβ-induced paralysis, had no effect on ROS intracellular levels in CL2006 worms [135]. Lastly, Indian gooseberry, velvet bean, pomegranate or turmeric extract increased H_2_O_2_-induced oxidative stress resistance in the CL4176 strain [114]. Despite the mentioned effects on the resistance to oxidative stress or ROS levels, oxidative damage markers were usually not evaluated. The unique study that did evaluate the oxidative damage markers reported lower protein oxidative damage in CL2331 and GMC101 worms treated with 2,3-dehydrosilybin A/B [126].

To protect themselves, cells have mechanisms to counteract ROS-induced damage, employing antioxidant enzymes and free radical scavengers such as superoxide dismutases (SOD) and catalases [162,163]. Therefore, the reduced levels of ROS and enhanced resistance to oxidative stress could be a consequence of increased enzymatic and non-enzymatic defense system activity. On one hand, this could simply be due to the cellular accumulation of different compounds with free radical scavenger activity from the tested products. However, it is also plausible that enzymatic defense was induced by the treatment. Five genes encoding for five isoforms with different subcellular localization have been described in the *C. elegans* genome. The subcellular localization of the different isoforms determines their efficacy against different ROS-inducing compounds. Catalase activity was measured only in animals treated with 2,3-dehydrosilybin A/B, and it was increased by the treatment [126]. In general, antioxidant enzyme activities have been scarcely measured in AD models treated with the mentioned products.

In the CL1553 strain that expresses the fusion protein *sod*-3::GFP, florescence levels indicated that SOD-3, a mitochondrial isoform of SOD, was up-regulated by phosphatidylserine [161], caffeic acid [147] and withanolide A [143], as well as guarana [115], strawberry [112] and carqueja [104] extracts. Moreover, a mixture of (+)-catechins, caffeine and procyanidins improved the response to juglone [130]. In contrast, SOD-3 was down-regulated in worms treated with Indian laburnum extract [98]. Although the protein level and the activity of SOD-3 were not measured in AD models, the expression of the gene encoding for this enzyme *sod-3* was reported to be up-regulated in CL4176 worms treated with caffeic acid [146], and in CL2006 worms treated with otophylloside B [138]. A similar finding was found in wild-type nematodes treated with withanolide A [143]. This confirmed that caffeic acid, at least, would have similar effects on different strains inducing SOD by up-regulating *sod-3* gene expression. If something similar occurred in transgenic AD models treated with the other compounds, SOD3 induction could be a key event for improving antioxidant defenses and protecting against oxidative stress. Nevertheless, studies confirming the importance of SOD3 in the protective effects of the compounds against changes in phenotype induced by Aβ are needed.

Lastly, the effects of some of the mentioned products on glutathione S-transferase 4 (GST-4) that could contribute to reduce the accumulation of damaged molecules have also been evaluated. In the CL2166 strain that expresses the fusion protein gst-4::GFP, it was confirmed that caffeic acid [147], withanolide A [143], Kushui rose essential oil [121] and the extracts of Tiew kon [127] guarana [115] and carqueja [104] up-regulated the levels of this protein. Likewise, *Gst-4* gene expression was up-regulated by caffeic acid treatment in CL4176 worms [147] and withanolide A in N2 nematodes [143]. However, Indian laburnum down-regulated GST-4 levels [98] and the treatment with otophylloside B had no effect on *gst-4* expression in the CL2006 strain [138].

### 6.5. Heat Stress Response

Endogenous chaperone proteins can also have an important role in the modulation of Aβ toxicity. Chaperone or chaperone-related proteins directly interact with Aβs and might play a role in some amyloid pathways, as well as in the protective responses in P*unc-54*::Aβ^1–42^ models. The reported effect on Aβ-induced paralysis supports that the interaction of these chaperone proteins with Aβ is part of a protective cellular response. Consistent with this observation, transgenic *C. elegans* displayed a significant decrease in Aβ toxicity and Aβ oligomers after supporting a heat shock (35 °C for 2 h), probably through the induction of heat shock proteins by heat shock transcription factor 1 (HSF-1) [164]. Importantly, an enhanced expression of human αB-crystallin, a well-studied stress-inducible chaperone protein, has been reported in AD patient brain tissues, indicating that proteins involved in heat stress response are also relevant for the pathology of the disease in humans [86].

The treatments with withanolide A [143], extracts of moringa [102] and guarana [115], and Japanese zelkova essential oil [122] increased the resistance to heat of the treated worms. However, caffeic acid [147] roselle extract and its constituent hydroxycitric acid [119] were unable to increase the mentioned resistance. It was also reported that caffeic acid had no effect on heat-induced ROS levels [147] in CL4176 nematodes, but the measurement of ROS under thermal stress is rare in this type of study.

Further studies that used transgenic *C. elegans* to identify the binding partners of Aβ have found that the chaperone protein HSP-16.2 has a protective effect against Aβ toxicity in vivo [164]. Gene expressions of *C. elegans* human αB-crystallin homologous HSP16-2 and HSP16-4 were up-regulated in transgenic CL4176 worms following a temperature up-shift [86]. In fact, the overexpression of both HSP-16.2 and human Hsp70 partially suppressed Aβ1–42 toxicity in a *C. elegans* AD model. Assays in the TJ375 or CL2070 strains, which expressed the fusion protein hsp-16.2::GFP, allowed the determination that treatment with phosphatidylserine [161], the extracts of Indian laburnum [98], *T. chebula* [101], carqueja [104], tiew kon [127], and guarana [115] led to an increase in HSP-16.2 levels. The treatment with caffeic acid showed a similar effect in TJ375 worms, but also led to up-regulated *Hsp-16.2* gene expression in CL2006 and CL4176 worms [147]. These data suggest that the increases in HSP-16.2 levels reported in TJ375 or CL2070 strains would also occur in *C. elegans* AD models, and probably in other strains. According to this observation, *Hsp-16.2* mRNA levels found in wild-type worms treated with withanolide A [143] and CL2006 nematodes treated with the extracts of yerba mate [110] and cranberry [116], would indicate that these treatments also increased the levels of this chaperone. In this last strain, a relative amount of *Hsp-16.2* mRNA-induced by thermal stress was also higher in tiew kon extract-treated animals [127]. However, the treatment with the extract of carqueja [104] had an antagonistic role in the effect of heat and juglone on HSP-16.2 levels. Likewise, haemanthidine from *L. radiata* reduced the gene expression of stress-related HSP-16 in CL4176 worms [149].

### 6.6. Transcription Factors Involved in Stress-Related Pathways

Stress-related pathways, that seem to play a role in the alleviation of AD pathology, are highly conserved in *C. elegans* [107,163,164]. These involve several transcription factors, such as DAF-16, HSF-1 and SKN-1. The insulin/IGF-1-mediated detoxification of Aβ aggregates requires both *daf-16* and *hsf-1*, which are downstream-regulated genes of the receptor gene *daf-2* [165]. Stress-related transcriptional factors DAF-16 and SKN-1, the homologous of mammal FOXO and Nrf2, have been found to be involved in Aβ detoxification. Assays carried out on the *C. elegans* strains expressing fusion proteins daf-16::GFP (TJ356) or skn-1::GFP (LD1 and LG333) support that extracts of strawberry [112] and *T. chebula* [101] induced the translocation of the transcription factors SKN-1 and DAF-16 to the nucleus. A similar effect on DAF-16 was confirmed for extracts of Indian laburnum [98], moringa [102] and roselle, as well as hydroxycitric acid [119], caffeic acid [147] and selenocysteine [166], but their effects on LD1 and LG333 strains were not evaluated. On the contrary, SKN-1 activation was also observed in worms treated with carqueja extract [104] or Kushui rose essential [121], but their effects were not tested on the TJ356 strain. However, no effect on DAF-16 was found for withanolide A [143] or Kushui rose essential [121], despite their effects against Aβ toxicity in the CL4176 and CL2355 strains, respectively. Therefore, it was necessary to confirm if the activation of DAF-16 and SKN-1 was involved in AD detoxification. When nematodes fed bacteria with RNAi of *daf-16* or *skn-1,* most of the effects on phenotypic changes were abolished or partially reduced. In this sense, it was reported that the effects of the Aβ-induced paralysis of the strawberry [112] and roselle extracts, as well as hydroxycitric acid [119] in the CL2006 and CL4176 strains, were *daf-16*- and *skn-1*-dependent. Similar consequences of iRNA treatment were reported for tiew kon- [127] and guarana-treated [115] nematodes, but their effects were not evaluated in strains expressing the fusion proteins daf-16::GFP or *skn-1*::GFP. The effects of moringa extract were also *daf-16*-dependent, but the role of *skn-1* was not explored [102]. Moreover, the effects of extracts of *T. chebula* [101], yerba mate and caffeine [110] on Aβ-induced paralysis were reported to be *daf-16*-dependent, but the effect of iRNA of *skn-1* was not tested in worms treated with these products. On the contrary, coffee effects [107] were found to be *skn-1*-dependent, but the effects of daf-16 RNAi in the experiments evaluating the anti-AD activities of this product were not. Among the tested products, the unique product with anti-AD effects that was confirmed to be independent of some of the mentioned transcription factors was Kushui rose essential oil, whose anti-AD activities in the CL4176 and CL2355 strains were *skn-1*-dependent and *daf-16*-independent [121]. This correlated with the lack of differences in DAF-16 cellular distribution between the control and Kushui rose essential oil-treated worms of the TJ356 strain. The decreased oligomerization of Aβ in CL2006 nematodes treated with this product was also demonstrated to be *skn-1*-dependent. Nevertheless, the iRNA of daf-16 was not tested in animals treated with the essential oil, since the effects on other phenotypic changes were *daf-16*-independent [121]. In addition, other experiments with iRNA showed that the 2,3-dehydrosilybin A/B effect on intracellular ROS levels and catalase activity in wild-type nematodes was *daf-16*-dependent, despite its effect on lifespan and pharyngeal pumping in wild-type nematodes [126] being *daf-16*-independent.

Heat-shock experiments showed that the expression of the genes *hsf-1* and *daf-16* also affected lifespan in *C. elegans* [167]. In wild-type nematode lifespan extension by phosphatidylserine [161], the extract of roselle or its constituent hydroxycitric acid [119] were *daf-16-* and *skn-1*-dependent. On the other hand, selenocysteine demonstrated that an increase in lifespan was *skn-1*-dependent, but the effect of iRNA of *daf-16* was not tested [166]. However, no effect on lifespan was found for holy basil extract, eugenol [168] or withanolide A [143] in *daf-16*-null nematodes, which is consistent with the increase in relative amounts of *daf-16* mRNA in N2 Bristol worms treated withanolide A [143]. The latter maintained its lifespan-extending effect on EU1 nematodes that were *skn-1*-null, indicating that its effect on longevity was also *skn-1*-independent. Altogether, these results suggest that the increase in the translocation of DAF-16 or/and SKN-1 observed in TJ356 worms would also occur in other *C. elegans* strains, including transgenic AD models. The activation of one or both transcription factors is presumably necessary for preventing or reducing Aβ transgene-associated phenotypic changes by the evaluated products. However, the activation of SKN-1 does not seem indispensable, although it could contribute to anti-AD activity.

Lastly, possible implications of the transcription factor HSF-1 in anti-AD activities shown by some natural products were also evaluated, although in a very reduced number of studies. The anti-AD activity of *T. chebula* extracts in CL4176 was demonstrated to also be *hsf-1*-dependent [101]. In this sense, a proantocyanidin-rich extract of cranberry [116] reduced Aβ-induced paralysis in CL2006 worms in a *hsf-1*-dependent, but *skn-1-* and *daf-16*-independent manner. In contrast, yerba mate extract and caffeine activities on Aβ-induced paralysis were confirmed to be *hsf-1*-independent [110]. However, HSF-1 activity was not evaluated.

Importantly, a mixture of (+)-catechins, caffeine and procyanidins [130] also led to an increase in juglone-induced DAF-16 translocation to the nucleus, suggesting that synergy between the tested products and stresses that activate the evaluated pathways is possible. Moreover, the expression of the *sir-2.1* gene that encodes an orthologue of human SIRT1 was also up-regulated, which mediates DAF-16 activation [130]. This protein was also involved in the effects of the previously mentioned products, but this role has not been analyzed in depth.

Some studies have confirmed that certain treatments were also able to modulate the expression of the genes encoding for the mentioned transcription factors. Among them, the expression of *hsf-1* gene was increased by caffeic acid in CL4176 [147] and withanolide A in N2 Bristol worms [143], which also led to an increase in *daf-16* gene expression, whereas the mixture of (+)-catechins, caffeine and procyanidins, tested in CL4176, had no effect on the expression of either gene [130]. However, the increase in the expression of these genes would not necessarily involve an increase in the translocation to the nucleus and the subsequent expression of genes with a promotor for these transcription factors, although the increased expression of downstream genes such as *sod-3, hsp-16.2* and *gst-4* was reported, as occurred in *C. elegans* treated with caffeic acid [147] or cranberry extract [116]. On the other hand, the expression of *daf-16* was not modulated by otophylloside B in the CL2006 strain [138] However, it was not necessary to modulate gene expression to increase activity of the transcription factors.

### 6.7. Other Chaperoness, Proteasome Activity and Autophagy

Lysosomes [169], mitochondria [170], and especially the endoplasmic reticulum (ER) [171] have been identified as targets for Aβs. Aβ aggregates can cause functional impairments in the organelles, but protein–protein interactions can also contribute to reduce their function [172]. Protein quality control systems can either repair or degrade damaged protein, preserving and maintaining protein-folding homeostasis (proteostasis). The correct three-dimensional structure of the proteins is indispensable for their functionality and, therefore, for the cellular functionality underlying organism health [172]. If these mechanisms do not adequately respond to proteotoxic imbalances, misfolded protein accumulation can lead to neurodegeneration. Proteostasis mechanisms involve the conservation of protein conformation or refolding by ER and mitochondrial unfolding protein response, as well as the degradation of proteins through proteasomal degradation or autophagy. This allows the removal of non-functional proteins in order to enable the synthesis of new functional proteins. Therefore, all of them could be implicated in reducing Aβ toxicity. HSP-16.2 has been shown to prevent unfolded proteins from aggregating and to interact with the intracellular human Aβ^1–42^ peptide [166,167]. This protein has been evaluated in the context of heat stress response. However, the effect of additional products with anti-AD activity in other elements of proteostasis networks and the implications of this in the prevention or reduction of Aβ^1–42^-induced phenotype changes has been little explored.

Increased proteasome activity and chaperone activation are among the highly conserved responses to maintain proteostasis [173,174]. In *C. elegans*, mitochondrial unfolding protein response participants include members of the major chaperone families, proteases and assembly factors [88]. Mitochondria-specific chaperones are encoded by *hsp-6* and *hsp-60* genes that encode for members of the HSP70 and HSP40 superfamilies [175]. Upon mitochondrial stress, the transcription factor DVE-1 binds to *hsp-6* and *hsp-60* promoters enhancing its expression [176]. To adequately develop its function, DVE-1 requires UBL-5 that seems to permit their interactions with cis-elements of the DNA [177]. It has been reported that *hsp-6*, *hsp-60* and *dve-1* can be involved in the prevention of Aβ^1–42^-induced paralysis. In fact, its knockdown increased the paralysis rate in AD models [141]. On the other hand, HSP-4 is an ER chaperone that plays a central role in the response to stress associated with both heat shock and protein misfolding [178]. XBP-1 is also a key factor to rescue stress resistance within the ER unfolded protein response [179] and has been proven to be necessary for the limitation of paralysis in AD models [140].

Some studies have evaluated the possible effects of natural products on the gene expression of proteins participating in unfolding protein responses. Treatments with otophylloside B [138] and cranberry extract [116] were proven to increase *hsp-12.6* and *hsp-70* gene expression in CL2006 nematodes. On the other hand, *P. gymnospora* extract and α-bisabolol induced *dnj-4* gene expression but down-regulated *hsp-4 gene* expression in CL2006 [123]. Knocking down experiments indicated that quercetin activity against Aβ-induced paralysis was independent of *hsp-6, hsp-60, dve-1* or *ubl-5,* as well as XBP-1 [147], although resveratrol effects depended on the mitochondrial unfolded protein response [142]. The differences in the chaperone and protease machineries of the mitochondria relative to the cytosol lead to questions on folding specificity and capacity. In particular, the mitochondrial protein quality control system is also essential to facilitate the translocation of mitochondrial proteins, assisting in their refolding and assembly. In fact, *hsp-6* has shown to be essential for the import of all nuclear encoded mitochondrial proteins avoiding abnormal mitochondrial morphology, lower ATP levels, defects in embryogenesis, and a shorter lifespan [180,181]. Experiments with a Huntingtin N-terminal fragment targeted to the mitochondria suggested that the subcellular compartment can have a strong effect on the equilibrium between folding and misfolding, which leads to the question of whether protein quality control in a specific compartment can influence proteotoxicity beyond its organellar boundary [182]. It has been proposed that Hsp70 specifically contributes to the cellular proteostasis network. In particular, this chaperone would have the most dramatic effect on the mitochondrial folding since it is the major mitochondrial chaperone. According to the last idea, it has been hypothesized that a reduction in the levels of Hsp70 should abolish the import of essential proteins into mitochondria and could, therefore, lead to an accumulation of mitochondrial pre-proteins in the cytosol [183].

The importance of the ubiquitin-proteasome system in AD was revealed when knocking down genes encoding for crucial factors of this system increased the paralysis of the nematodes [140]. The in vitro activity of 26S proteasomes isolated from wild-type nematodes treated with carqueja extract was higher than from the control individuals [104]. Proteasomal activity also was elevated in quercetin-treated CL2006 worms [145], suggesting that some compounds could promote the removal of misfolding proteins in wild-type animals, but also in AD models. Moreover, anti-AD effects of quercetin including both Aβ deposits and Aβ-induced paralysis were reported to depend on proteasomal protein degradation.

Autophagy presents in different forms, but all of them can contribute to cytosolic Aβ aggregate removal. Chaperone-mediated autophagy is a type of autophagy that does not require the formation of vesicles because cytosolic proteins are directly transferred into the lysosomal lumen by chaperones. Another, less selective type is macroautophagy, which is featured by the formation of an autophagosome and subsequent fusion with lysosomes [184]. The knockdown of *bec-1*, which encodes an essential component for autophagosome formation [185], led to an increase in paralysis observed in AD *C. elegans* models. A reduction in Aβ deposits and paralysis delay in CL4176 by resveratrol [142] or quercetin [145] were confirmed to depend on macroautophagy. Interestingly, lysosome amount was reduced in CL2006 nematodes treated with quercetin and CL4176 nematodes treated with resveratrol, which could be a consequence of an elevated autophagic flux. Moreover, pharmacological inhibition studies have indicated that the decrease in Aβ-induced paralysis in CL4176 worms caused by ferulic acid was dependent on autophagy [146]. Functional DEGs enrichment analysis of the whole transcriptome sequencing after temperature up-shifting in ferulic-acid-treated worms showed that this compound modulated longevity, ErbB, mTOR, the TGF-beta signaling pathway, the ribosome, and the phagosome-referencing KEGG pathways. The down-regulated gene RSK-1 (ribosomal protein S6 kinase beta) seemed to be responsible for the change in longevity, ErbB, mTOR, and the TGF-beta signaling pathway. Thus, RSK-1 was associated with autophagy since it modulated mTOR activity [186]. Moreover, in the HLH-30 strain, the protein HLH-30, the highest homology to mammalian transcription factor EB (TFEB) that regulates both autophagy and lysosomal lipolysis function, was activated by a similar treatment [187,188,189,190,191]. Moreover, the promotion of autophagosome formation by ferulic acid treatment has been confirmed in the DA2123 strain [146] that contains the fusion protein GFP:LGG-1 [192]. Likewise, in CL4176 worms, caffeic acid up-regulated the expression of the *lgg-1* gene that is involved in autophagy [147]. Moreover, Western blots allowed the determination that this compound increased the flux of the autophagic pathway [146]. The staining of lysosomes in CL2006 worms treated with quercetin [145] also suggested that this compound promotes lysosomal protein degradation, even under unfavorable conditions. On the other hand, ferulic acid also reduced the level of lipids in a dose-dependent manner in CL4176 nematodes [146]. Since bacterial intake was maintained, it was suggested that the activation of autophagy and lipolysis by ferulic acid was a fasting-like response.

Regarding chaperone-mediated autophagy, two lysosomal-associated membrane glycoprotein homologs, *lmp-2* and *unc-46,* have been identified. The reduction in paralysis by quercetin was not affected by RNAi for either of them, suggesting that the anti-AD activity of this compound would not require chaperone-mediated autophagy [145]. In contrast, the protective effect against Aβ-induced paralysis of resveratrol was evidenced to be dependent on chaperone-mediated autophagy, but also macroautophagy [142].

All the mentioned effects of natural products on different proteins patircipating in autophagy, proteostasis, cell siganling pathways and antioxidant defenses are listed in Table 4.

### 6.8. Inflammation

Different pro-inflammatory genes and cytokines have been associated with human neurodegenerative diseases, contributing to pathology. *C. elegans* contains two homologues of TNFα-induced protein 1 (TNFA1P1) encoded by the *F22E5.6* and *ZC239.12* genes, the expression of which were up-regulated in transgenic CL4176 upon temperature up-shift [86]. In particular, TNFA1P1 expression was found to be increased in AD patient brain tissues [86]. Although TNFA1P1 has not been associated with Aβ toxicity in humans [86], it has been more widely evidenced that TNF-α signaling exacerbates both Aβ and tau pathologies in vivo [193,194], although some researchers have reported that the treatment with TNFα can protect hippocampal human neurons cultures exposed to Aβ [195]. The expression of an inflammation-associated gene, the TNFA1P-homolog F22E5.6, in CL4176 worms treated with galanthamine, haemanthidine or 1,2-Di-O-acetyllycorine (all isolated from *L. radiata*) was reduced [149]. This effect could contribute to reduced cell damage, but additional inflammation-related markers should be explored in this model.

### 6.9. Acetyl-Choline Metabolism in Neuronal Synapsis

AD is a disorder where the cholinergic system is most severely compromised. In fact, the principal mechanism behind available and accepted therapies for AD is the use of AChE inhibitors, which lead to an elevation of Ach levels [196,197]. Some tested natural products could also result in being useful for modulating the Ach metabolism and neuronal cholinergic synapses. Thyme oil and its components thymol and para-cymene decreased AchE activity in N2 Bristol *C. elegans* leading to increased Ach synaptic levels [132]. Yerba mate extract and caffeine also reduced AChE activity in CL2006 [110]. Interestingly, thyme oil and para-cymene even increased the responsiveness of nAChR [132]. Unlike mammals with only one AChE gene [196], *C. elegans* have multiple *ace* genes that are expressed in different regions [197,198]. The analysis of the expression of some *ace* genes also suggested a reduction in AChE activity in animals treated with certain natural products. In this sense, galanthamine and haemanthidine decreased *ace-1* and *ace-2* expression in CL4176 nematodes after the temperature up-shift [149], whereas *P*. *gymnospora* extract and its constituent α-bisabolol only down-regulated the expression of *ace-1* in the same strain [123]. However, not all product reducing or preventing phenotypic changes associated with the presence of the transgene in AD *C. elegans* models necessarily modulated AChE. For instance, the treatment with cranberry extract had no effect on *act-1* gene expression in the CL2006 strain [116].

## 7. Conclusions and Future Perspectives

The present review aimed to explore the targets shared by natural products investigating AD treatment or prevention, based on research using *C. elegans* as a model organism. In recent years, a wide variety of natural products have been tested in transgenic *C. elegans* models, reflecting the great potential of this model for the discovery of neuroprotective products. Most of the tested product showed certain protective effects in terms of phenotypic changes, including both behavioral changes and the accumulation of protein aggregates. These assays reveal that phenotypic screening continues to be very useful for discovering new bioactive compounds. The possible effects of oxidative stress have been explored, but it remains to be elucidated if this is truly involved in the initiation and progression of the disease, or if it is a consequence. Other aspects studied include the role of cell signaling pathways. In this sense, the use of strains with knocked out genes has helped, but the use of RNAi offers more interesting advantages because it allows the direct investigation of the role of these pathways in AD models. In contrast, inflammation-related mechanisms, mitochondrial biology, neurotransmitter imbalances and proteostasis mechanisms, including unfolded protein response participants and autophagy, were investigated in a small number of studies. Since all of these are said to play a role in the pathology of AD, the interest and increase in the number of studies evaluating them is clear. On the other hand, except for one study, all research has been based on the *C. elegans* expression of human Aβs, so there is a bias towards the amyloid pathway. Therefore, it is necessary to perform new assays in new models of tauopathy, considering the failures in the clinical trials of drugs targeting this pathway and the reported associations between NFT levels and cognitive function. In addition, there are a lack of studies evaluating the possible effects of products after the pathological process has started, and it would be very interesting to explore the possibility of reversing the disease, at least in its earlier phases. Finally, it is important to underline the differences between humans and *C. elegans,* from pathology to the metabolism of the tested products. For this reason, research based on *C. elegans* should be complementary, rather than an alternative approach to the use of mammals. Therefore, the evidence obtained from worms must be validated in mammalian systems.

Lastly, it is important to consider that curcumin, EGCG, gossypol and resveratrol have been considered nuisance compounds [199] or “pan assay interference compounds (PAINS)” by Baell et al. [200,201,202], because such compounds frequently hitter in biological assays causing important problems in drug discovery research. This is due to these compounds modulating bioactivity through mechanisms of action considered undesirable because they generally cause cellular injury, but with an inherent lack of specificity. Consequently, they would interfere with cell assay results. The possible “undesirable” mechanisms exerted by PAINS include nonspecific electrophilicity, colloidal aggregation, redox cycling, membrane perturbation and chelation. The cellular injury derived from the mentioned mechanisms is in line with the concept “cytotoxicity burst”. This phenomenon occurs at relatively high compound concentrations and implies a set of cellular activities that are thought to result from the activation of multiple stress responses as opposed to originating from the activation of a specific molecular target [203]. As shown, many compounds evaluated in AD *C. elegans* models activate cell signaling pathways involved in certain stress responses, which could be a consequence of cellular injury. Moreover, many of the tested natural products contain a mixture of substances which can share substructures with the PAINS identified to date. In addition, some of the substances present in them are uncharacterized. Therefore, many natural products are susceptible to interference by all the aforementioned mechanisms of action; therefore, these products should be explored in future studies. Likewise, testing the effects of these products at multiple concentrations, as well as the prefractionation of the extracts, have been proposed as potential solutions to these problems [202].

## Figures and Tables

**Table 1:** Main transgenic Alzheimer’s disease *C. elegans* models.

Strain (Promoter::Transgene)	Transgene Expression	Phenotype
CL2006 (P*unc-54*::SP::Aβ^1–42^)	Constitutive expression in muscle cells	Progressive, adult-onset paralysis. Intramuscular Aβ deposits.
CL2122 (P*unc-54*::SP::Aβ^1–42^)	Constitutive expression in muscle cells	Slow adult movement. Intramuscular Aβ deposits.
CL4176 (P*myo-3*::SP::Aβ^1–42^::long 3′ UTR)	Inducible expression in muscle cells	Temperature-inducible larval paralysis. Intramuscular Aβ deposits.
CL2355 (P*snb-1*::SP:Aβ^1–42^::long 3′UTR)	Inducible pan-neuronal expression	Memory deficits, abnormal thrashing in liquid, partial sterility.

Abbreviations: Aβ—β amyloid peptide; SP—signal peptide.

**Table 2:** Beneficial effect of natural products on features of Alzheimer’s disease and pathology-related processes.

Effect on Pathology	Strain	Extract/Essential Oil	Ref.
Lifespan/survival increase	N2 Bristol wild type	*Cassia fistula* L. (Indian laburnum)	[98]
		“barquillo”, by-product of (*Theobroma cacao* L.) cocoa	[125]
		*Hibiscus sabdariffa* L. (roselle)	[119]
		*Jasonia glutinosa* (L.) DC. (rock tea)	[109]
		*Ilex paraguariensis* A.St.-Hil. (yerba mate)	[110]
		Leaf of *Moringa oleifera* Lam. (moringa)	[102]
		Fruit of *Paullinia cupana* Kunth (guarana)	[115]
		Silymarin, extract of seeds of *Silybum marianum* (L.) Gaertn. (*milk thistle*)	[126]
	CL4176	*Ocimum sanctum* L. (holy basil)	[117]
Aβ-induced paralysis reduction	CL2006	Leaf of *Cocos nucifera* L. (Coconut)	[124]
		*Cratoxylum formosum ssp. Pruniflorum* (Tiew kon) twig	[127]
		*Glochidion zeylanicum* (Gaertn.) A. Juss.	[100]
		*Ilex paraguariensis* A.St.-Hil. (yerba mate)	[110]
		*Padina gymnospora* (Kütz.) Sond. (Funnelweed)	[123]
		Fruit of *Paullinia cupana* Kunth (guarana)	[115]
		*Sideritis scardica* Griseb. (Greek mountain tea)	[108]
		*Terminalia chebula* Retz.	[101]
		*Punica granatum* (pomegranate)	[114]
	GMC101	Root and rhizome of *Salvia miltiorrhiza* Bunge (Danshen)	[128]
	CL4176	*Baccharis trimera* (Less) DC (Carqueja)	[104]
		*Camelia sinensis* (tea)	[129]
		*Camellia sinensis* var. Kitamura (Zijuan Pu’er tea)	[130]
		*Cassia fistula* L. (Indian laburnum)	[98]
		Leaf of *Cocos nucifera* L. (Coconut)	[124]
		*Curcuma longa* (turmeric)	[114]
		seed of *Coffea arabica* L. (decaffeinated Coffee), Decaffeinated	[107]
		*Glochidion zeylanicum* (Gaertn.) A. Juss.	[100]
		*Hibiscus sabdariffa* L. (roselle)	[119]
		*Jasonia glutinosa* (L.) DC. *(rock tea)*	[109]
		Leaf of *Moringa oleifera* Lam. (moringa)	[102]
		Seeds of *Mucuna pruriens* (L.) DC. (velvet bean)	[98]
		*Padina gymnospora* (Kütz.) Sond. (Funnelweed)	[123]
		Fruit of *Paullinia cupana* Kunth (guarana)	[115]
		Fruits of *Phyllanthus emblica* L. (Indian gooseberry)	[103]
		Essential oil of *Rosa setate × Rosa rugosa* (Kushui rose)	[121]
		*Sideritis scardica* Griseb. (Greek mountain tea)	[108]
		*Fragaria × ananassa* (Strawberry)	[112]
		*Terminalia chebula* Retz.	[101]
		*Zelkova serrata* (Thunb.) Makino (Japanese zelkova)	[122]
		*Viola cornuta* (horned violet)	[120]
		*Viola x wittrockiana* (pansy)	
Improvement of Aβ-induced chemotaxis defects	CL2355	*Ginkgo biloba* leaf	[99]
		*Glochidion zeylanicum* (Gaertn.) A. Juss.	[100]
		*Lignosus rhinocerus* (Tiger Milk Mushroom)	[131]
Learning improvement	CL2355	*Acorus tatarinowii* (Schott)	[105]
Serotonin sensitivity increase	CL2355	*Acorus tatarinowii* (Schott)	[105]
		*Ginkgo biloba* leaf	[99]
		*Rosa setate × Rosa rugosa* (Kushui rose)	[121]
Reduction in Aβ deposits	CL2006	Leaf of *Cocos nucifera* L. (Coconut)	[124]
		*Ginkgo biloba* leaf	[99]
		Roots of *Glycyrrhiza uralensis* Fisch.(Chinese liquorice)	[97]
		*Rosa setate × Rosa rugosa* (Kushui rose)	[121]
		*Sideritis scardica* Griseb. (Greek mountain tea)	[108]
	CL4176	*Acorus tatarinowii* (Schott) oil	[105]
		Leaf of *Cocos nucifera* L. (Coconut)	[124]
		*Padina gymnospora* (Kütz.) Sond. (Funnelweed)	[123]
		*Sideritis scardica* Griseb. (Greek mountain tea)	[108]
		*Fragaria × ananassan* (Strawberry)	[112]
	CL2355	*Acorus tatarinowii* Schott	[105]
	CL2179	*Ginkgo biloba* leaf extract	[99]
	GMC101	Root and rhizome of *Salvia miltiorrhiza* Bunge (Danshen)	[128]
Aβ oligomerization reduction	CL2006	*Rosa setate × Rosa rugosa* (Kushui rose)	[121]
		*Terminalia chebula* Retz.	[101]
	CL2355	*Ginkgo biloba* leaf extract	[99]
Increase in synaptic levels of acetyl-choline	N2 Bristol wild type	Thymus vulgaris (Thyme)	[132]
Reduction in ROS levels	N2 Bristol wild type	*Cassia fistula* L. (Indian laburnum)	[98]
		*Hibiscus sabdariffa* L. (roselle)	[119]
		Hydrolyzed cocoa by-product	[125]
		*Ilex paraguariensis* A.St.-Hil. (yerba mate)	[110]
		Leaf of *Moringa oleifera* Lam. (moringa)	[102]
		*Fragaria × ananassa* (Strawberry)	[112]
	CL2006	Cratoxylum formosum ssp. Pruniflorum (Tiewkon) twig	[127]
		Leaf of *Ginkgo biloba* L.	[99]
		*Padina gymnospora* (Kütz.) Sond. (Funnelweed)	[123]
		Fruit of *Paullinia cupana* Kunth (guarana)	[115]
	BA17 wild type	*Baccharis trimera* (Less) DC (Carqueja)	[104]
	GMC101	Root and rhizome of *Salvia miltiorrhiza* Bunge (Danshen)	[128]
Reduction in chemically induced ROS	N2 Bristol wild type	Fruit of *Paullinia cupana* Kunth (guarana)	[115]
Resistance to induced oxidative stress improvement	N2 Bristol wild type	*Cassia fistula* L. (Indian laburnum)	[98]
		Hydrolyzed cocoa by-product	[127]
		*Ilex paraguariensis* A.St.-Hil. (yerba mate)	[110]
		*Jasonia glutinosa* (L.) DC. *(rock tea)*	[109]
		Fruit of *Paullinia cupana* Kunth (guarana)	[115]
		*Zelkova serrata* (Thunb.) Makino (Japanese zelkova)	[122]
		*Viola cornuta* (horned violet)	[120]
		*Viola x wittrockiana* (pansy)	
		Leaf of *Ginkgo biloba* L.	[99]
	CL4176	*Curcuma longa* (turmeric)	[114]
		*Phyllanthus emblica* (Indian gooseberry)	
		Fruits of *Punica granatum* L. (pomegranate)	
	BA17 wild type	*Baccharis trimera* (Less) DC (Carqueja)	[104]
Resistance to thermal stress improvement	N2 Bristol wild type	Leaf of *Moringa oleifera* Lam. (moringa)	[102]
		Fruit of *Paullinia cupana* Kunth (guarana)	[115]
		*Zelkova serrata* (Thunb.) Makino (Japanese zelkova)	[122]
Improvement in healthspan parameters	N2 Bristol wild type	*Hibiscus sabdariffa* L. (roselle)	[119]
		Leaf of *Moringa oleifera* Lam. (moringa)	[102]
		Fruit of *Paullinia cupana* Kunth (guarana)	[115]

Abbreviations: ROS—reactive oxygen species; Aβ—β-amyloid.

**Table 3:** Beneficial effect of compounds isolated from natural products on features of Alzheimer’s disease and pathology-related processes.

Effect on Pathology	Strain	Compound	Source	Ref.
Lifespan/survival increase	N2 Bristol wild type	2,3-dehydrosilybin A/B	Silymarin, extract of seeds of *Silybum marianum* (L.) Gaertn. (*milk thistle*)	[126]
		20-galloyl-dehydrosilybin A/B		
		3,7-dimethyl- dehydrosilybin A/B		
		7-methyl- dehydrosilybin A/B		
		Isosilybin A		
		Silybin A/B		
		Silychristin A/B		
		Silydianin A/B		
		Caffeic acid	Various	[146]
		Caffeine	*Coffea arabica* L. (Coffee); *Camelia sinensis* (L.) Kuntze (tea)	[110]
		Hydroxycitric acid	*Hibiscus sabdariffa L.* (roselle)	[119]
		Peptides	Hydrolyzed “barquillo”, by-product of (*Theobroma cacao* L.) cocoa	[125]
		Sesamin	Seeds of *Sesamum indicum* (sesame)	[134]
		Withanolide A	Root of *Withania somnifera* (L.) Dunal (ashwagandha)	[143]
	CL2006	Ferulic acid	Various	[140]
		Morin	Various	
		Quercetin	Various	
		Gossypol	*Gossypium arboreum* L. (Cotton plant)	
		Oleuropein aglycone	Extra virgin oil form Olea europaea L. fruit (olive oil)	[135]
	CL2355	Cannabidiol	*Cannabis sativa* L. (marijuana)	[148]
	CL4176	Caffeic acid	Various	[147]
		Eugenol	*Ocimum sanctum* L. (holy basil)	[117]
		Haemanthidine	*Lycoris radiata* (L’Hér.) Herb. *(red spider lily)*	[149]
	GMC101	6‴-feruloylspinosin	Seeds of *Ziziphus jujuba* Mill. (Sour Jujube)	[150]
Aβ-induced paralysis reduction	CL2006	Cyclotides	*Clitoria ternatea* L. (butterfly pea)	[141]
		Quercetin	Various	[144,145]
		Oleuropein aglycone	Extra virgin oil form Olea europaea L. fruit (olive oil)	[135]
		Otophylloside B	*Cynanchum otophyllum* L. (dog-strangling vine)	[138]
		Resveratrol	Red wine	[142]
		Betalains	Rhizome of *Beta vulgaris* L. (Red-beet)	[139]
	CL4176	Withanolide A	Root of *Withania somnifera* (L.) Dunal (ashwagandha)	[143]
		Mixture of (+)-catechins, caffeine and procyanidins	Various	[130]
		Peptides	Hydrolyzed “barquillo”, by-product of (*Theobroma cacao* L.) cocoa	[125]
		2,3-dehydrosilybin A	Silymarin, extract of seeds of *Silybum marianum* (L.) Gaertn. (*milk thistle*)	[126]
		2,3-dehydrosilybin B		[126]
		Aglycon of glycyrrhizic acid	Roots of *Glycyrrhiza uralensis* Fisch.(Chinese liquorice)	[97]
		Glycyrrhetinic acid		
		Glycyrrhizic acid		
		Isoliquiritigenin		
		Liquiritigenin		
		Caffeic acid	Various	[147]
		7-hydroxycalamenene	Essential oil of *Zelkova serrata* (Thunb.) Makino *(Japanese zelkova)*	[122]
		Caffeine	*Coffea arabica* L. (Coffee); *Camelia sinensis* (L.) Kuntze (tea)	[107]
		Cyclotides	*Clitoria ternatea* L. (butterfly pea)	[141]
		Ferulic acid	Various	[146]
		Ginkgolide A	Leaf of *Ginkgo biloba* L.	[99]
		Ginkgolide J		
		Haemanthidine	*Lycoris radiata* (L’Hér.) Herb. *(red spider lily)*	[149]
		Nardochinins A	Underground parts of *Nardostachys chinensis* Batal. (Chinese Nardostachys)	[133]
		Nardochinins B		
		Nardochinins D		
		Hydroxycitric acid	*Hibiscus sabdariffa L.* (roselle)	[119]
		Imbricatolic acid	Seeds of *Platycladus orientalis* (L.) Franco (Chinese arborvitae)	[103]
		Isocupressic acid		
		Magnolol	*Magnolia* spp.	[136]
		Nardochinin B	Underground parts of *Nardostachys chinensis* Batal. (Chinese Nardostachys)	[133]
		Nardochinins A		
		Nardochinins C		
		Nardochinins D		
		Nubiol	*Salvia castanea* Diels	[137]
		Salcastanins A-F		
		Oleuropein aglycone	Extra virgin oil form *Olea europaea* L. fruit (olive oil)	[135]
		Otophylloside B	*Cynanchum otophyllum L. (dog-strangling vine)*	[138]
		Sesamin	Seeds of *Sesamum indicum* (sesame)	[134]
	GMC101	2,3-dehydrosilybin A/B	Silymarin, extract of seeds of *Silybum marianum* (L.) Gaertn. (*milk thistle*)	[126]
		6‴-feruloylspinosin	Seeds of *Ziziphus jujuba* Mill. (Sour Jujube)	[150]
Improvements in Aβ-induced chemotaxis defects	CL2355	Caffeic acid	Various	[147]
		Cannabidiol	*Cannabis sativa* L. (marijuana)	[148]
		Cyclotides	*Clitoria ternatea* L. (butterfly pea)	[141]
		Otophylloside B	*Cynanchum otophyllum* L. (dog-strangling vine)	[138]
		Sesamin	Seeds of *Sesamum indicum* (sesame)	[134]
		Sesamolin		
	GMC101	6‴-feruloylspinosin	Seeds of *Ziziphus jujuba* Mill. (Sour Jujube)	[150]
Learning improvements	CL241	Magnolol	*Magnolia* sp.	[136]
	GMC101	6‴-feruloylspinosin	Seeds of *Ziziphus jujuba* Mill. (Sour Jujube)	[150]
Serotonin sensitivity increase	CL2355	Ferulic acid	Various	[146]
		Geraniol	Essential oil of *Rosa setate × Rosa rugosa* (Kushui rose)	[121]
		Ginkgolide A	Leaf of *Ginkgo biloba* L.	[99]
Reduction in Aβ deposits	CL2006	Aglycon of glycyrrhizic acid	Roots of *Glycyrrhiza uralensis* Fisch.(Chinese liquorice)	[97]
		Glycyrrhetinic acid		
		Glycyrrhizic acid		
		Isoliquiritigenin		
		Cyclotides	*Clitoria ternatea* L. (butterfly pea)	[141]
		Ferulic acid	Various	[146]
		Oleuropein aglycone	Extra virgin oil form *Olea europaea* L. fruit (olive oil)	[135]
		α-bisabolol	*Padina gymnospora* (Kütz.) Sond. (Funnelweed)	[123]
	CL2331	2,3-dehydrosilybin A/B	Silymarin, extract of seeds of *Silybum marianum* (L.) Gaertn. (*milk thistle*)	[126]
	CL4176	2,3-dehydrosilybin A		[144]
		2,3-dehydrosilybin B		
		Silybin B		
		α-bisabolol	*Padina gymnospora* (Kütz.) Sond. (Funnelweed)	[123]
Aβ oligomerization reduction	CL2006	Quercetin	Various	[146]
		Oleuropein aglycone	Extra virgin oil form Olea europaea L. fruit (olive oil)	[135]
	CL4176	Ferulic acid	Various	[146]
		Ginkgolide A	Leaf of *Ginkgo biloba* L.	[99]
		Ginkgolide J		
		Hydroxycinnamic acid	Various	[151]
		Sesamin	Seeds of *Sesamum indicum* (sesame)	[134]
	GMC101	2,3-dehydrosilybin A/B	Silymarin, extract of seeds of *Silybum marianum* (L.) Gaertn. (*milk thistle*)	[126]
Increase in synaptic levels of acetyl-choline	N2 Bristol wild type	2,3-dehydrosilybin A/B	Silymarin, extract of seeds of *Silybum marianum* (L.) Gaertn. (*milk thistle*)	[126]
		Gamma-terpinene	Essential oil of *Thymus vulgaris* L. (thyme)	[132]
		para-Cymene		
		Thymol		
		Withanolide A	Root of *Withania somnifera* (L.) Dunal (ashwagandha)	[143]
Reduction in ROS levels	N2 Bristol wild type	2,3-dehydrosilybin A/B	Silymarin, extract of seeds of *Silybum marianum* (L.) Gaertn. (*milk thistle*)	[126]
		Caffeine	*Coffea arabica* L. (Coffee); *Camelia sinensis* (L.) Kuntze (tea)	[110]
		Hydroxycitric acid	*Hibiscus sabdariffa L.* (roselle)	[119]
		Withanolide A	Root of *Withania somnifera* (L.) Dunal (ashwagandha)	[143]
	CL2006	Caffeine	Coffee, tea	[110]
		Cyclotides	*Clitoria ternatea* L. (Butterfly pea)	[141]
		Kaempherol	Various	[106]
		Ascorbate	Various	
		Otophylloside B	*Cynanchum otophyllum L. (dog-strangling vine)*	[138]
		α-bisabolol	*Padina gymnospora* (Kütz.) Sond. (Funnelweed)	[123]
	CL4176	7-hydroxycalamenene	Essential oil of *Zelkova serrata* (Thunb.) Makino *(Japanese zelkova)*	[122]
		Cyclotides	*Clitoria ternatea* L. (Butterfly pea)	[141]
		Ginkgolide A	Leaf of *Ginkgo biloba* L.	[152]
		α-bisabolol	*Padina gymnospora* (Kütz.) Sond. (Funnelweed)	[123]
Reduction in chemically induced ROS		Caffeic acid	Various	[147]
		Withanolide A	Root of *Withania somnifera* (L.) Dunal (ashwagandha)	[143]
Resistance to induced oxidative stress increase	N2 Bristol wild type	2,3-dehydrosilybin A/B	Silymarin, extract of seeds of *Silybum marianum* (L.) Gaertn. (*milk thistle*)	[126]
		Caffeine	*Coffea arabica* L. (Coffee); *Camelia sinensis* (L.) Kuntze (tea)	[110]
		Withanolide A	Root of *Withania somnifera* (L.) Dunal (ashwagandha)	[143]
Oxidative damage accumulation reduction	CL2331	2,3-dehydrosilybin A/B	Silymarin, extract of seeds of *Silybum marianum* (L.) Gaertn. (*milk thistle*)	[126]
	GMC101			
Resistance to thermal stress increase	N2 Bristol wild type	Caffeic acid	Various	[147]
		Withanolide A	Root of *Withania somnifera* (L.) Dunal (ashwagandha)	[143]
Improvements in healthspan parameters	N2 Bristol wild type	2,3-dehydrosilybin A/B	Silymarin, extract of seeds of *Silybum marianum* (L.) Gaertn. (*milk thistle*)	[126]
		Withanolide A	Root of *Withania somnifera* (L.) Dunal (ashwagandha)	[143]
	CL2355	Cannabidiol	*Cannabis sativa* L. (marijuana)	[148]

Abbreviations: ROS—reactive oxygen species; Aβ—β-amyloid.

**Table 4:** Effects of natural products on different proteins and relevance for Alzheimer´s disease pathology.

Protein	Effect	Strain	Extract/Compound	iRNA Effects on AD Models Treated with the Extract/Compound	Ref.
SOD-3	Up-regulated levels	CL1553	Phosphatidylserine		[159]
			Caffeic acid		[147]
			Withanolide A		[143]
			Guarana extract		[115]
			Strawberry extracts		[112]
			Carqueja extract		[104]
	Up-regulated gene expression	N2 Bristol	Withanolide A		[143]
		CL4176	Caffeic acid		[147]
			Cranberry extract		[116]
	Down-regulated levels		Indian laburnum extract		[98]
GST-4	Up-regulated levels	CL2166	Withanolide A		[143]
			Caffeic acid		[147]
			Rose essential oil		[123]
			Guarana extract		[115]
			Carqueja extract		[106]
			Tiew kon extract		[127]
	Up-regulated gene expression		Caffeic acid		[147]
			Cranberry extract		[116]
	Down-regulated levels	CL2166	Indian laburnum extract		[98]
	No effect on gene expression	CL2006	Otophylloside B		[138]
HSP-16.2	Up-regulated levels	TJ375 or CL2070	Phosphatidylserine		[159]
			Guarana extract		[115]
			Carqueja extract		[106]
			Indian laburnum		[98]
			*T. chebula* extract		[101]
	Up-regulated gene expression	N2	Withanolide A		[143]
		CL2006	Yerba mate extract		[110]
			Cranberry extract		[116]
		CL2006	Caffeic acid		[147]
		CL4176	Caffeic acid		[147]
			Cranberry extract		[116]
SKN-1	Translocation to the nucleus	LD1 and LG333	Strawberry extract	Aβ-induced paralysis was partially reduced by iRNAs	[112]
			Carqueja extract		[104]
			Kushui rose essential oil		[121]
	Unknown		Kushui rose essential oil	No consequences of iRNAs treatment in CL4176 and CL2355	[121]
			Proantocyanide-rich extract of cranberry	No consequences of iRNAs treatment in CL2006	[116]
			Coffee	Aβ-induced paralysis was partially reduced by iRNAs	[107]
			Guarana extract		[115]
			Tiew kon extract		[127]
DAF-16	Translocation to the nucleus	TJ356	Strawberry extract	Aβ-induced paralysis was partially reduced by iRNAs	[112]
			roselle extract		[119]
			*T. chebula* extract		[101]
			Moringa extract		[102]
			Selenocysteine		[166]
			Caffeic acid		[147]
			Indian laburnum extract		[98]
			Hydroxycitric acid		[119]
	No effect on translocation to nucleus	TJ356	Withanolide A		[143]
			Kushui rose essential oil		[121]
			Kushui rose essential oil.	No consequences of iRNAs treatment in CL4176 and CL2355 strains	[121]
			Proantocyanide-rich extract of cranberry	No consequences of iRNAs treatment in CL2006	[116]
	Up-regulated gene expression	CL4176	Caffeic acid		[147]
		N2 Bristol	Withanolide A		[143]
	No effect on gene expression	CL4176	Mixture of (+)-catechins, caffeine and procyanidins		[130]
	Unknown		Tiew kon extract	Aβ-induced paralysis were partially reduced by iRNAs	[127]
			Guarana extract		[115]
			Yerba mate extract		[110]
			Caffeine		
HSF-1	Unknown	CL4176	*T. chebula* extracts	Aβ-induced paralysis was partially reduced by iRNAs	[101]
		CL2006	Proantocyanide-rich extract of cranberry		[116]
			Yerba mate extract	No consequences of iRNAs treatment in CL4176 and CL2355 strains	[110]
			Caffeine		[110]
	Up-regulated gene expression	CL4176	Caffeic acid		[147]
		N2 Bristol	Withanolide A		[143]
	No effect on gene expression	CL4176	Mixture of (+)-catechins, caffeine and procyanidins		[130]

Abbreviations: SOD-3—superoxide dismutase 3; GST-4—glutathione-S-transferase 4; HSP-16.2—heat shock protein 16.2; HSF-1—heat shock factor-1; Aβ—β-amyloid.
